# The chordata olfactory receptor database

**DOI:** 10.1093/procel/pwae050

**Published:** 2024-09-20

**Authors:** Wei Han, Siyu Bao, Jintao Liu, Yiran Wu, Liting Zeng, Tao Zhang, Ningmeng Chen, Kai Yao, Shunguo Fan, Aiping Huang, Yuanyuan Feng, Guiquan Zhang, Ruiyi Zhang, Hongjin Zhu, Tian Hua, Zhijie Liu, Lina Cao, Xingxu Huang, Suwen Zhao

**Affiliations:** iHuman Institute, ShanghaiTech University, Shanghai 201210, China; Research Center for Life Sciences Computing, Zhejiang Lab, Hangzhou, Zhejiang 311121, China; Department of Intelligent Edge Cloud, China Telecom Cloud Technology Co., Ltd., Shanghai 200120, China; Department of Intelligent Edge Cloud, China Telecom Cloud Technology Co., Ltd., Shanghai 200120, China; iHuman Institute, ShanghaiTech University, Shanghai 201210, China; School of Life Science and Technology, ShanghaiTech University, Shanghai 201210, China; iHuman Institute, ShanghaiTech University, Shanghai 201210, China; School of Life Science and Technology, ShanghaiTech University, Shanghai 201210, China; iHuman Institute, ShanghaiTech University, Shanghai 201210, China; School of Information Science and Technology, ShanghaiTech University, Shanghai 201210, China; Department of Intelligent Edge Cloud, China Telecom Cloud Technology Co., Ltd., Shanghai 200120, China; Department of Intelligent Edge Cloud, China Telecom Cloud Technology Co., Ltd., Shanghai 200120, China; Department of Intelligent Edge Cloud, China Telecom Cloud Technology Co., Ltd., Shanghai 200120, China; Department of Intelligent Edge Cloud, China Telecom Cloud Technology Co., Ltd., Shanghai 200120, China; Research Center for Life Sciences Computing, Zhejiang Lab, Hangzhou, Zhejiang 311121, China; Research Center for Life Sciences Computing, Zhejiang Lab, Hangzhou, Zhejiang 311121, China; iHuman Institute, ShanghaiTech University, Shanghai 201210, China; School of Life Science and Technology, ShanghaiTech University, Shanghai 201210, China; iHuman Institute, ShanghaiTech University, Shanghai 201210, China; School of Life Science and Technology, ShanghaiTech University, Shanghai 201210, China; iHuman Institute, ShanghaiTech University, Shanghai 201210, China; School of Life Science and Technology, ShanghaiTech University, Shanghai 201210, China; Shanghai Key Laboratory of High-Resolution Electron Microscopy, ShanghaiTech University, Shanghai 201210, China; iHuman Institute, ShanghaiTech University, Shanghai 201210, China; School of Life Science and Technology, ShanghaiTech University, Shanghai 201210, China; Shanghai Key Laboratory of High-Resolution Electron Microscopy, ShanghaiTech University, Shanghai 201210, China; Department of Intelligent Edge Cloud, China Telecom Cloud Technology Co., Ltd., Shanghai 200120, China; Research Center for Life Sciences Computing, Zhejiang Lab, Hangzhou, Zhejiang 311121, China; Zhejiang Provincial Key Laboratory of Pancreatic Disease, The First Affiliated Hospital, and Institute of Translational Medicine, Zhejiang University School of Medicine, Hangzhou 310029, China; iHuman Institute, ShanghaiTech University, Shanghai 201210, China; School of Life Science and Technology, ShanghaiTech University, Shanghai 201210, China; Shanghai Key Laboratory of High-Resolution Electron Microscopy, ShanghaiTech University, Shanghai 201210, China; Shanghai Clinical Research and Trial Center, Shanghai 201210, China

## Abstract

**Graphical Abstract:**
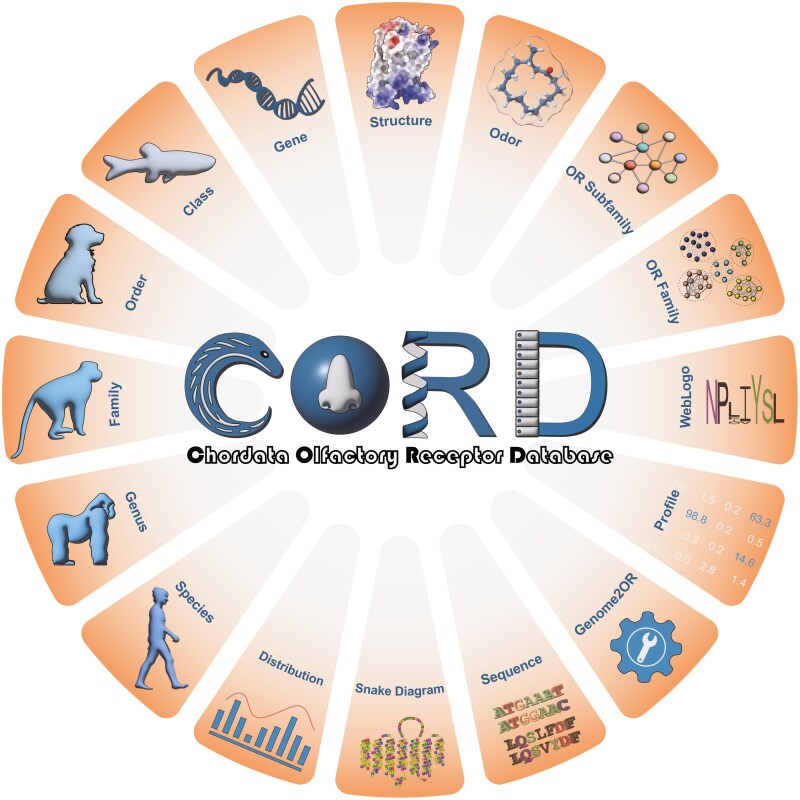

## Introduction of database

Olfaction is one of the oldest chemosensory systems in chordates, playing crucial roles in their foraging, predator evasion, social communication, mating and parental care (Guo et al., 2023; Li and Liberles, 2015; Liberles, 2014). The initial step of olfaction is the binding and activation of olfactory receptors (ORs) by odorants in a combinatorial way (Malnic et al., 1999). In chordates, ORs are encoded by OR genes (*olfrs*). Generally, *olfrs* are expressed on the cilia of olfactory sensory neurons, more and more *olfrs* have been found to be ectopically expressed in various non-olfactory tissues and some of them are disease-related (Buck and Axel, 1991; Drew, 2022; Massberg and Hatt, 2018). For example, Orecchioni et al. had shown that the octanal receptors Olfr2 in mouse and OR6A2 in human vascular macrophages, upon interaction with toll-like receptor 4, initiate inflammasome activation in the presence of octanal, which in turn leads to the production and secretion of IL-1α and IL-1β proteins (Orecchioni et al., 2022). This response, along with the involvement of other inflammatory cytokines, is likely responsible for the pronounced effects of Olfr2 observed in mouse models of atherosclerosis (Orecchioni et al., 2022). Cheng et al. elucidated the binding mechanism of the insulin peptide insB:9-23 to the OR Olfr109 and demonstrated that a pepducin-based antagonist targeting Olfr109 ameliorated glucose homeostasis in mouse models of diabetes and obesity (Cheng et al., 2022). This suggests that ORs not only form the basis of olfactory function but also hold significant importance in understanding chordate biology and advancing disease treatments.

In chordates, the rapid evolution of *olfrs* has been suggested to be influenced by the unique living environments of specific species (Nei et al., 2008; Niimura, 2009). This diversity is evident in the varying number of *olfrs* among species: the African elephant genome encodes around 2,000 functional ORs, compared to about 1,200 in mouse, approximately 400 in humans, and just over 100 in zebrafish (Han et al., 2022; Niimura et al., 2014). Additionally, most species’ genomes include a high-proportion of OR pseudogenes. In humans, for instance, the genome contains over 400 OR pseudogenes, accounting for about 50% of the total *olfrs* (Han et al., 2022). These factors collectively increase the complexity of annotating *olfrs* based on sequence homology. Furthermore, the quality of genome sequencing significantly impacts the accuracy of *olfr* annotation. Poor sequencing and assembly quality, such as sequencing errors, incomplete sequences, or misjoins, can lead to erroneous annotations of *olfrs*. In fact, only a few species have had their *olfrs* thoroughly annotated, with data primarily recorded in six chordate OR-related databases: ORDB (Marenco et al., 2013), OlfactionDB (Modena et al., 2011), ODORactor (Liu et al., 2011), HORDE (Olender et al., 2013), OlfactionBase (Sharma et al., 2022), and M2OR (Lalis et al., 2024). However, with advancements in sequencing technology and reductions in sequencing costs, approximately 3,000 chordate species have now had their whole genomes sequenced, many of which are of high-quality (Satam et al., 2023; Sayers et al., 2023). However, the OR data for most species have not been effectively annotated or systematically integrated.

To address the lack of effective annotation and systematic integration of OR data across numerous species, we developed Genome2OR. This tool, which is based on a hidden Markov model (HMM) of DNA sequences, provides a rapid and sensitive solution for annotating *olfrs* from genomes (Han et al., 2022). Recently, we updated the Genome2OR to simplify the annotation process and employed it to perform exhaustive annotations of thousands of chordate genomes, yielding over one million *olfrs*. Leveraging these annotations, along with a multitude of derived datasets and additional external data we curated, we created the chordata olfactory receptor database (CORD, website of CORD). CORD is an online resource that offers a rich and comprehensive collection of OR data. With its user-friendly interface and an intelligent, efficient underlying database system, users can easily access and retrieve the data they need. We believe that CORD will be a valuable resource for the research community in the OR field, and it will also engage the broader public interested in the science of olfaction.

## Overview of database

CORD offers a comprehensive and high-quality resource for chordate ORs, covering a wide range of data from species diversity to the molecular level. It features a user-friendly interface and a robust underlying database system, ensuring that users can easily access, analyze, and retrieve the information they need.

CORD integrates data from 2,776 species across seven major evolutionary clades: lancelets, jawless fish, jawed fish, amphibians, reptiles, birds, and mammals, and includes 1,176,818 *olfrs* (Table 1). These genes comprise 663,380 functional *olfrs* and 513,438 OR pseudogenes, showcasing the depth and breadth of the data (Table 1). Furthermore, CORD features data on odorants, OR–odorants pairs and high-precision protein models (Ahdritz et al., 2024), snake diagrams (Isberg et al., 2016), BLAST (Camacho et al., 2009), profiles, WebLogos (Crooks et al., 2004), and sequence similarity networks, providing robust support for structural and functional studies of ORs.

**Table 1.** Overview of databases related to chordate ORs.

Database	Species	Func. *olfrs*	Pseu. *olfrs*	Pairs	Odors	Year
ORDB	70	18,735		547	95	2000
ODORactor	2	1,516	92	636	3,038	2011
OlfactionDB	2	83		400	85	2012
HORDE	11	6,736	4,336			2013
OlfactionBase	2	150		874	330	2022
M2OR	11	1,246		3,108	768	2023
GPCRdb	1	27	0	24	24	2023
CORD	2,776	663,380	513,438	3,118	23,690	2024

In the table, the ‘Database’ column indicates the names of the listed databases; the ‘Species’ column reflects the number of species with OR records in each database; the ‘Func. *olfrs*’ column records the number of functional ORs in each database; the ‘Pseu. *olfrs*’ column records the number of OR pseudogenes; the ‘Pairs’ column denotes the number of OR-odorant pairs recorded in the database; the ‘Odors’ column shows the number of odorants recorded in the database; the ‘Year’ column specifies the year the database was established.

The user interface of CORD is designed to be intuitive, offering a user-friendly querying experience. Interactive data visualizations and advanced search options enhance the efficiency of data retrieval and analysis. Notably, CORD emphasizes data localization and cross-referencing, allowing users to download the entire dataset while offering extensive links to both internal and external databases. This feature facilitates in-depth exploration and cross-database applications, providing users with comprehensive research capabilities.

In summary, CORD significantly enhances the accuracy and convenience of data retrieval and analysis by expanding the coverage of OR data in chordates and optimizing data visualization and user interface design. This provides robust data support for research in olfactory science and related biomedical fields.

## CORD interface and navigation

**Figure 1.**
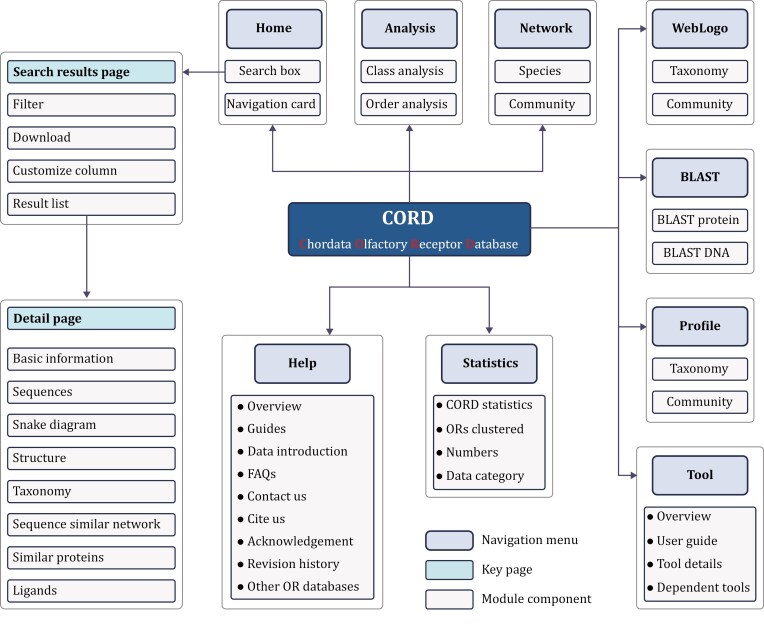
The database architecture of CORD. This figure provides a detailed overview of the user interface architecture of the CORD, including various main functional modules and navigation components.

CORD is an open-access web server offering free data browsing and download services. The platform features nine functional menus: Home, BLAST, WebLogo, Profile, Network, Analysis, Tool, Statistics, and Help (Fig. 1). Additionally, it includes two key information display pages: the search results page and the detail page. With its intuitive interface and robust search functionality, users can easily access, analyze, and retrieve the OR data they need (Fig. 1).

### Home menu: intuitive navigation and advanced search features

**Figure 2.**
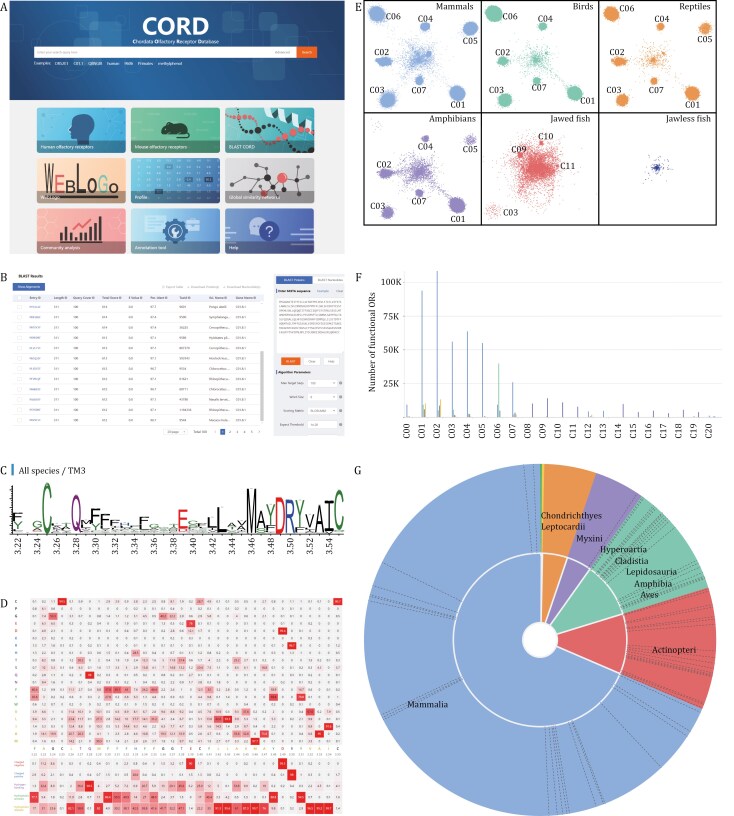
Menus of CORD. The figure displays the following menus of CORD: (A) ‘Home’, (B) ‘BLAST’, (C) ‘WebLogo’, (D) ‘Profile’, (E) ‘Network’, (F) ‘Analysis’, and (G) ‘Statistics’.

The Home menu provides intuitive access to information through the search box and navigation cards, enabling users to quickly retrieve data (Fig. 2A). The search box features a built-in fuzzy matching algorithm that enables rapid searches across 18 fields. For users requiring more precise queries, the “Advanced” button next to the search box offers access to advanced search options, allowing for exact or fuzzy searches based on logical combinations in specific fields, thus meeting detailed query needs. The navigation cards serve as shortcuts to popular sections of the database, simplifying user navigation. The design of the search box and navigation cards fully embodies CORD’s user-centric design philosophy, ensuring that both new users and experienced researchers can efficiently browse the database.

### Search result page: personalized search result customization

In CORD, user-initiated searches are directed to a well-designed search results page. This page intuitively displays search results in a table format, featuring up to 35 customizable attribute fields such as “Organism,” “Gene Name,” and “UniProt ID,” providing a multidimensional data view. Users can sort, filter, and customize the display fields and their order according to their personal needs, achieving a personalized data interface. The filtering panel on the page supports various filtering criteria, enhancing search accuracy. Additionally, users can easily download search results in different file formats, facilitating data export and analysis.

### Detail page: comprehensive OR information display

The detail page is the core component of CORD for presenting OR information, with each OR having its dedicated page. The page comprises eight modules that comprehensively introduce receptor information. The “basic information” module provides an overview of essential OR data; the “Sequences” module displays protein and DNA sequences in a color-coded format and includes links to the BLAST menu; the “snake diagram” module highlights the main structural features of the OR, using color coding for enhanced recognition (Pandy-Szekeres et al., 2023); the “structure” module offers structural prediction results and model quality assessments, including predicted local distance difference test, predicted aligned error, and multiple sequence alignment (Varadi et al., 2024); the “taxonomy” module presents basic species information and OR distribution; the “sequence similarity network” module displays the sequence similarity network among ORs within a species; the “similar proteins” module lists similar proteins based on sequence identity; and for ORs with OR–odorant pairs data, the detail page also features a “ligands” module, which illustrates the relationships between ORs and ligands. The detail page not only displays comprehensive data but also enhances interactivity and data export functions, enabling researchers to easily access, analyze, and share information.

### BLAST menu: homology sequence search

The BLAST menu integrates homology sequence search functionality based on the NCBI blast + toolkit (v2.15.0) (Camacho et al., 2009) (Fig. 2B). Users can submit query sequences to search for similar protein or DNA sequences within the CORD, utilizing the BLAST menu’s powerful search capabilities. This page provides flexibility, enabling users to adjust key parameters of the BLAST algorithm, such as *E*-value, word size, and scoring matrix, to optimize search results according to their research needs (Camacho et al., 2009). Additionally, the download and export functions of the BLAST page greatly streamline data retrieval and subsequent analysis.

### WebLogo menu: sequence conservation analysis

The WebLogo menu leverages the WebLogo (v3.7.8) tool to provide users with an intuitive interface for visualizing sequence conservation patterns across different OR families or species evolutionary lineages (Crooks et al., 2004). The page displays sequence conservation through stacked letters, with letter sizes representing residue frequency, where larger letters indicate higher conservation (Fig. 2C). Users can customize the view of conservation patterns based on specific structural regions, OR families and species evolutionary lineages. The page also supports the insertion of multiple WebLogos, enabling visual comparisons of conservation patterns across different OR families or species evolutionary lineages.

### Profile menu: residue distribution revealing

The Profile menu and WebLogo menu collectively undertake the task of analyzing conservation patterns within OR families (Fig. 2D). Although these two functional modules differ in their modes of representation, they both aim to uncover common features and patterns within OR families. The profile menu provides specific numerical values for residue distribution at each position, offering more detailed information, albeit in a less intuitive format compared to WebLogo (Fig. 2C).

### Network menu: OR network explorer

The Network menu offers an interactive network visualization tool for exploring the complex relationships between ORs (Fig. 2E). Users can select specific OR families or species using the control panel and adjust the style and color of data points. This tool simplifies the browsing and analysis of OR networks, thus aiding users in gaining a deeper understanding.

### Analysis menu: mapping the distribution of ORs

The tools in the Analysis menu assist researchers in exploring the distribution of ORs (Fig. 2F). By selecting species evolutionary lineages using the toggle panel, researchers can use histograms and heatmaps to visualize OR distribution. Histograms provide detailed distributions across different species or receptor families, while heatmaps offer a quick overview of overall distribution patterns.

### Tool menu: accessing and utilizing the annotation tool

The tool menu aids users in comprehensively understanding and utilizing Genome2OR, an OR genome annotation tool we developed (Han et al., 2022). This menu provides workflow diagrams, user guides, algorithm processes, and dependency lists, ensuring that users can easily download, install, and efficiently use the tool. These resources help users grasp the tool’s algorithmic details and effectively apply it in their genomic annotation and analysis research.

### Statistics menu: database data overview

The Statistics menu provides users with comprehensive statistics and visual representations of the database data (Fig. 2G). Through pie charts, histograms, and tables, users can quickly grasp the basic data overview of CORD. Additionally, this page showcases the data growth trajectory from previous versions to the current version, offering a clear comparison of data volume and highlighting the continuous improvement in the content and data quality of CORD.

### Help menu: comprehensive support hub

The Help menu is a comprehensive support center designed to help users thoroughly comprehend and efficiently use the database. It offers sections such as guides, data introduction, FAQs, contact us, cite us, acknowledgments, and revision history. These resources enable users to fully leverage the functionalities and resources of CORD.

## Comparison with previous work

CORD significantly advances the field of OR databases, improving data richness, species coverage, and technical architecture. Compared to our previous work, this update includes over 60% more data. Additionally, CORD introduces new data on odorants, OR–odorant pairs, high-precision protein models, and conceptual translation sequences of OR pseudogenes.

In a comparative analysis with other existing chordate OR databases, CORD demonstrates significant advantages across key metrics (Table 1). Compared to ORDB, the number of functional ORs in CORD has increased more than 35-fold, and species coverage has expanded from 70 to 2,776 species, representing nearly a 40-fold increase (Table 1). Regarding pseudogenes, although ODORactor and HORDE databases provide related data, CORD exhibits a clear advantage in the inclusion of OR pseudogenes. GPCRdb is undeniably a leading resource in the G protein-coupled receptor field, and we are enthusiastic users of this database. The comprehensive content and intuitive user interface of GPCRdb have been a significant source of inspiration for us, driving the development of CORD. While GPCRdb provides detailed and professional data on most human G protein-coupled receptors, it largely overlooks ORs, offering only limited information on a few dozen human ORs (Table 1). Additionally, CORD records 3,118 OR–odorant pairs and 23,690 odorants, the highest among all databases, providing a rich chemical and molecular foundation for the functional study of ORs.

## Database construction

### Update on genomic data acquisition for CORD

CORD continues the automated genomic data download process that was initiated in our previous work, ensuring a seamless continuation of data acquisition and integration (Han et al., 2022). This update focuses on the collection of incremental data, specifically all chordate genomic data published through the NCBI Assembly database from January 2021 to April 2023 (Taxon ID: 7711). Utilizing the NCBI Datasets tool (v15.10.0), we have newly downloaded 1,048 chordate genomic datasets, bringing the total number of species in CORD to 2,776 (Sayers et al., 2023).

### Updates and optimizations of Genome2OR

We have implemented significant updates and enhancements to the Genome2OR annotation tool, aimed at elevating the efficiency and automation of genome annotation. The new version of Genome2OR simplifies the annotation process by rewriting sections of the code, merging tasks that previously required three separate steps into a single step (Han et al., 2022). Furthermore, the new version incorporates the HMM Profile into the code, allowing users to swiftly invoke it by specifying parameters, thereby streamlining the annotation process. In addition, to accommodate users’ needs for customized HMM Profiles, we have retained an input interface that enables users to upload their customized HMM Profile files for personalized annotation. This improvement not only boosts the usability of Genome2OR but also provides users with greater flexibility.

### Conceptual translation of pseudogenes using FASTA suite

We employed the fasty tool from the FASTA suite to perform the conceptual translation of pseudogenes (Pearson, 2016). This process relies on a carefully curated and deduplicated target database that integrates OR protein sequences from nearly 3,000 species, ensuring its uniqueness and comprehensiveness. By matching the query DNA sequences against the protein database, we were able to identify the closest reference sequences, significantly enhancing the accuracy of the conceptual translation. Thanks to the richness and diversity of our database, we have successfully achieved conceptual translation for 99% of the pseudogenes in CORD.

### Implementation of protein sequence clustering for CORD

We implemented protein sequence clustering for CORD, using the same method as the Uniclust database, which is widely recognized in the industry for its efficiency and sensitivity (Jumper et al., 2021; Mirdita et al., 2017). We used the uniclust-pipeline (github.com/soedinglab/uniclust-pipeline) for protein sequence clustering, with core commands from the MMseqs2 (Mirdita et al., 2021) and HHsuite3 (Steinegger et al., 2019) suites. To accommodate the specific needs of CORD, we made necessary modifications to the original process code, especially in terms of clustering sensitivity and sequence identity threshold parameters. We chose more sensitive clustering parameters, for example, adjusting the ‘-s’ parameter in the mmseqs prefilter command from 1 to 6 to enhance the sensitivity of prefiltering, although this requires more computational resources. In addition, based on the classification knowledge of the OR family, we provided customized settings for the sequence consistency threshold parameters (Olender et al., 2020). By applying sequence consistency thresholds of 30%, 40%, 50%, 60%, 80%, and 90%, we successfully generated a series of clustering datasets from CORDclust30 to CORDclust90. It is worth noting that our clustering analysis not only covers functional OR protein sequences but also includes sequences obtained from the conceptual translation of OR pseudogenes.

### High-precision protein structure prediction of ORs with OpenFold

We employed the OpenFold tool to perform high-precision protein structure prediction on the 663,380 functional ORs present in our database (Ahdritz et al., 2024). Our workflow adhered to a stringent set of bioinformatics standards, ensuring the accuracy and reliability of the prediction process. Initially, we identified candidate sequences with sequence homology to the target ORs by conducting a sequence search on the CORDclust50 database using the HMMsearch algorithm (Eddy, 2011). Subsequently, these candidate sequences were realigned in a MSA the MAFFT software to ensure the high-accuracy of the alignment results (Katoh and Standley, 2013; Nakamura et al., 2018). Following this, we used the HHalign tool in the HHsuite3 toolkit to convert the alignment results from FASTA format to A3M format, meeting the input requirements of OpenFold (Steinegger et al., 2019). Ultimately, these standardized MSAs were input into OpenFold, where our advanced deep learning model facilitated protein structure prediction.

### OR community detection and network embedding in CORD

Initially, we performed MSAs of 663,380 functional OR protein sequences from the CORD and calculated their similarities, thereby constructing a global OR sequence similarity network (Han et al., 2022). Subsequently, we employed the parallel Louvain method algorithm from the NetworKit toolkit for community detection within this network (Angriman et al., 2022; Christian and Henning, 2016). Additionally, we utilized the LargeVis algorithm to project the network, reducing its 663,380 dimensions to 3 dimensions to enable visualization and analysis (Tang et al., 2016). Ultimately, we partitioned the functional ORs in CORD into 20 distinct communities.

### Data collection in CORD

To build the CORD database, we collected various types of data from multiple reliable sources, including odorants and their properties, OR–odorant pairs data, species lineage data, and *olfr* names. Notably, we obtained 76 attribute data of 23,690 odorants from The Good Scents Company (TGSC) and the PubChem database (Creek; Kim et al., 2023). Simultaneously, by integrating and analyzing seven databases—ORDB (Marenco et al., 2013), OlfactionBase (Sharma et al., 2022), M2OR (Lalis et al., 2024), OlfactionDB (Modena et al., 2011), ODORector (Liu et al., 2011), InAct (Del et al., 2022), and Glass (Chan et al., 2015), we obtained 3,118 OR–odorant pairs data entries after redundancy removal. To ensure the accuracy of species lineage data, we employed the TaxonKit tool for matching and downloading (Shen and Ren, 2021). Additionally, we obtained data such as *olfr* names from UniProt, adding important information dimensions to the CORD (UniProt Consortium, 2023).

### Web implementation of CORD

The web implementation of CORD adopts a front-end and back-end separation development concept, which allows the front-end user interface and back-end server logic to be developed and deployed independently. The user interface design of CORD is inspired by the UniProt database, aiming to provide a clear, intuitive, and professional navigation layout to ensure that users can efficiently access and analyze data (UniProt Consortium, 2023). The front-end uses the Vue.js (v3.3) framework and Element Plus (v2.3) component library to implement a responsive and componentized user interface (Kyriakidis et al., 2016). The back-end chooses Nginx (v1.21) as the web server and reverse proxy, combined with the module packaging optimization of Webpack (v5.88), ensuring the stability, security, and fast page loading time of the application (Reese, 2008). At the database level, CORD uses the NebulaGraph (v3.6) graph database, optimizing graph data storage and query performance, especially suitable for handling complex bioinformatics networks. The integration of Elasticsearch (v8.13) provides powerful full-text search capabilities, while MySQL (v8.0) ensures the consistency and integrity of transactional data (Gormley and Tong, 2015; Workbench, 2019). In terms of data visualization, by integrating AntV G2 (v4.2) and ECharts (v5.4) graphics libraries, CORD provides a wealth of data visualization options, not only enhancing the readability of data but also supporting researchers in deeply understanding complex bioinformatics data (Li et al., 2018).

## Future development

The CORD is dedicated to continuously integrating various data related to chordate ORs, aiming to serve scientists and the public interested in olfactory research. With the release of the first experimentally determined chordate OR structure (Billesbolle et al., 2023) and several consensus chordate OR structures (Choi et al., 2023), our understanding of OR structures has entered a new stage. Clearly, more and more OR structures are on their way. In the future, we plan to integrate experimental structural information of ORs into the CORD. Meanwhile, we plan to use new molecular simulation technologies such as AlphaFold3 to build odor–OR complex structures and put them into our database (Abramson et al., 2024). Additionally, we will continue updating new ORs from newly sequenced chordate genomes, introduce a more systematic classification system for ORs based on DNA phylogeny, and retain multiple previous classification systems to enable scientists to delve into the evolutionary relationships of these two major classes of ORs (Niimura et al., 2014; Olender et al., 2020). Functionally, we look forward to adding a genome browser feature in future versions of CORD, enabling users to analyze *olfrs* and their neighboring genes on the genome.

## Conclusion

CORD is a comprehensive and highly integrated OR database. This update has achieved significant growth in data volume and has made important advances in data types, user interface experience, and backend architecture performance. Through the carefully designed web interface and advanced search algorithms, CORD greatly enhances the efficiency of users in retrieving and analyzing data, providing a powerful tool for OR research.

Our database has achieved a 64% increase in the number of functional ORs, with the total number of functional genes reaching 663,380. We have also expanded species coverage by 64%, bringing the total number of species to 2,776, and added key data types such as odorants, OR–odorants and protein models. These rich data resources, combined with our continuous optimization of the Genome2OR tool, provide a solid foundation for cross-species comparative research. Furthermore, the backend architecture of CORD has been thoroughly restructured, adopting the NebulaGraph database and Elasticsearch full-text search engine, significantly improving search efficiency and intelligence.

We believe that the launch of CORD will benefit researchers and the public in the field of ORs. At the same time, we look forward to the addition of a genome browser feature in future versions, which will further enhance the database’s analytical capabilities and provide researchers with deeper insights.

## Data Availability

CORD is freely accessible at the website.

## Code availability

Genome2OR is freely available at the official website.
